# Supplementary material for: Neutralizing negative epigenetic regulation by HDAC5 enhances human haematopoietic stem cell homing and engraftment
Source: Nat Commun. 2018 Jul 16;9:2741. doi: 10.1038/s41467-018-05178-5 (PMC6048146; doi:10.1038/s41467-018-05178-5)
Supplement: Supplementary file 1 — Supplementary Information [file 41467_2018_5178_MOESM1_ESM.pdf]

Supplementary Table 1 SRC frequency determined by limiting dilution assay in NSG mice

Culture conditions	Cells transplanted	Number of mice with > 1% human cell chimerism/total number of mice
Vehicle control	500	0/5
	2500	5/5
	10000	4/5
M344	500	2/5
	2500	5/5
	10000	5/5

Supplementary Table 2 SRC frequency in vehicle or M344 treated group

Culture conditions	SRC frequency	95% confidence interval	Number of SRC in 1×10^6 $CD34^+$ cells
Vehicle control	1/3216	1/7392 to 1/1400	311
M344	1/746	1/1900 to 1/293	1341

SRC frequency was calculated by Poisson statistics from the data provided in Table S1 using ELDA software.

Supplementary Table 3 Determination of SRC frequency in vehicle or LMK235 treated group

Culture conditions	Cells transplanted	Number of mice with > 1% human cell chimerism/total number of mice
Vehicle control	500	0/5
	2500	1/5
	10000	4/5
LMK235	500	2/5
	2500	4/5
	10000	5/5

Supplementary Table 4 SRC frequency in vehicle or LMK235 treated group

Culture conditions	SRC frequency	95% confidence interval	Number of SRC in 1×10^6 $CD34^+$ cells
Vehicle control	1/7916	1/19377 to 1/3234	126
LMK235	1/1326	1/3219 to 1/546	754

SRC frequency was calculated by Poisson statistics from the data provided in Table S3 using ELDA software.

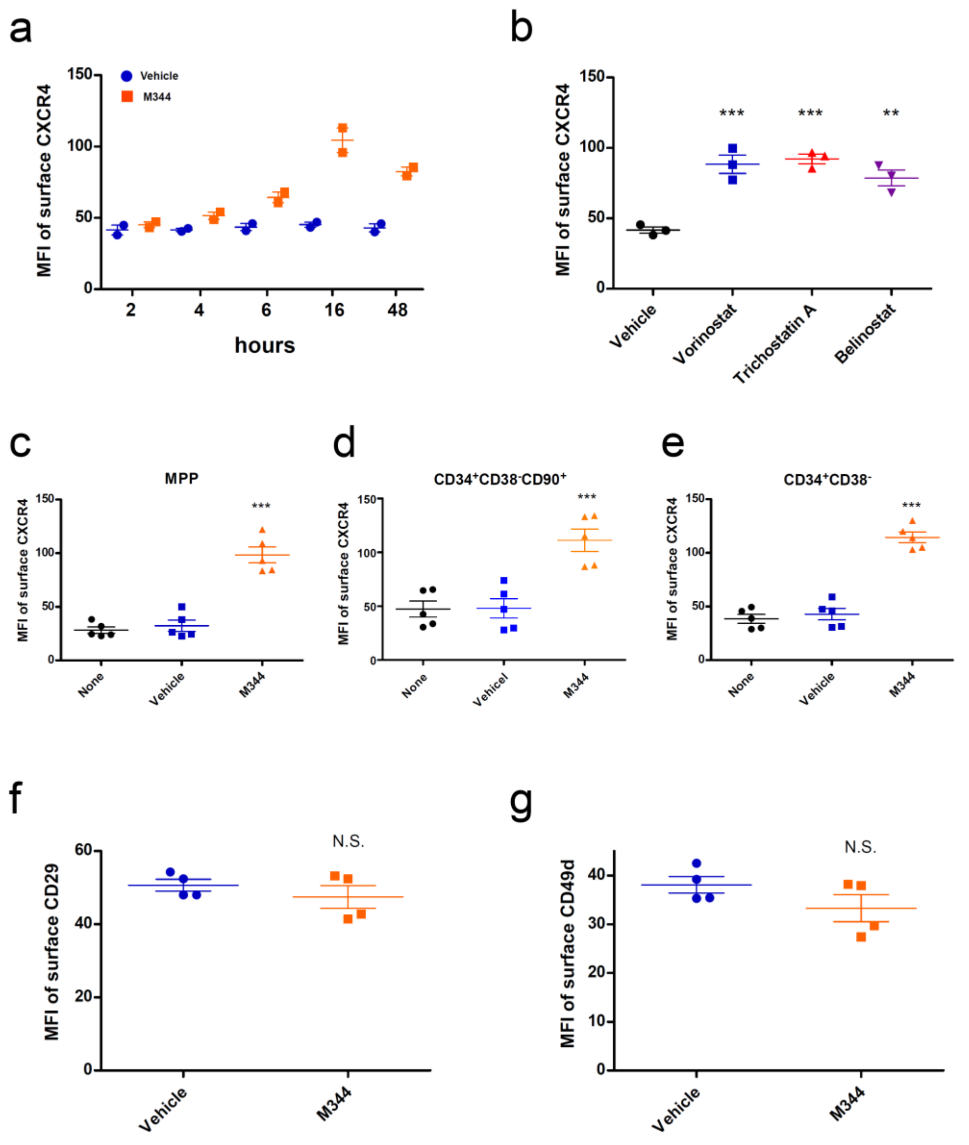

Supplementary Figure 1. Inhibition of HDAC promotes CXCR4 expression

(**a**) Mean fluorescence intensity (MFI) of surface CXCR4 of human CB CD34$^+$ cells treated with vehicle or HDAC inhibitor M344 for different times. Data pooled from two independent experiments are shown (n=2). (**b**) MFI of surface CXCR4 of human CB CD34$^+$ cells after treating cells for 16 hours with vehicle or different HDAC inhibitors. Data pooled from three independent experiments are shown (n=3, one-way ANOVA). (**c**) Quantification of MFI of surface CXCR4 of human CB MPPs (CD34$^+$CD38$^-$CD45RA$^-$CD90$^-$CD49f$^-$) treated with vehicle or M344. None indicates the group without any treatment. Data pooled from five independent experiments are shown (n=5, one-way ANOVA). (**d**) Quantification of MFI of surface CXCR4 of CD34$^+$CD38$^-$ cells treated with vehicle or M344. None indicates the group without any treatment. Data pooled from five independent experiments are shown (n=5, one-way ANOVA). (**e**) Quantification of MFI of surface CXCR4 of CD34$^+$CD38$^-$CD90$^+$ cells treated with vehicle or M344. None indicates the group without any treatment.

Data pooled from five independent experiments are shown (n=5, one-way ANOVA). (**f**) Quantification of MFI of surface CD29 of human CB human CB CD34^{+} cells treated with vehicle or M344. Data pooled from two independent experiments are shown (n=4). (**g**) Quantification of MFI of surface CD49d of human CB human CB CD34^{+} cells treated with vehicle or M344. Data pooled from two independent experiments are shown (n=4). Data are shown as dot plots (mean±s.e.m.). For all panels, **p<0.01. ***p<0.001. N.S. indicates not significant.

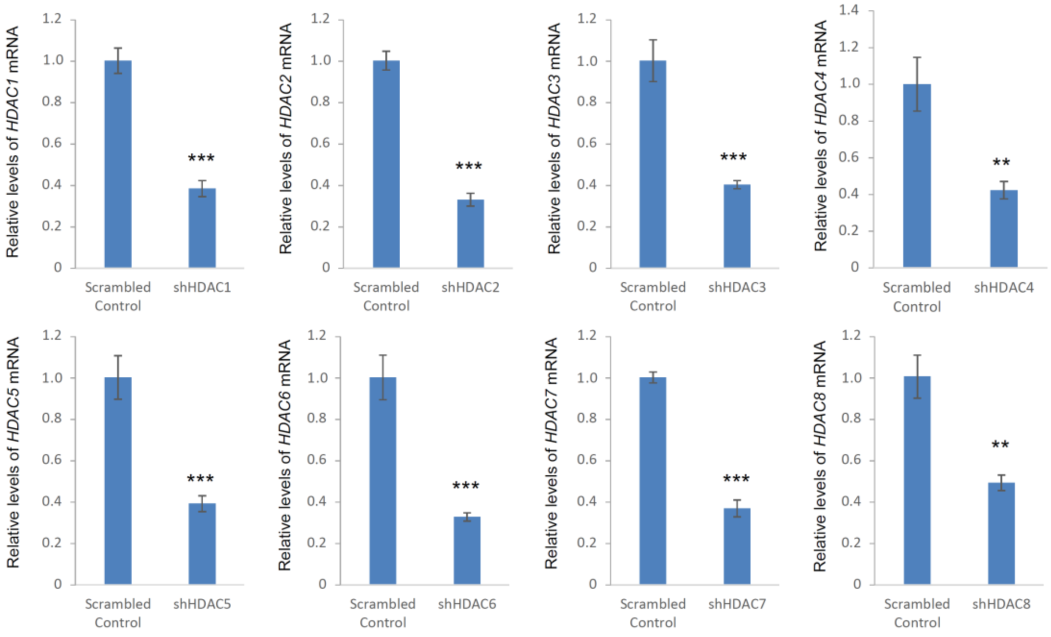

Supplementary Figure 2. mRNA levels of different HDACs after shRNA knockdown Individual shRNA constructs targeting different HDACs were transfected into 293T cells and cultured for 48 hours. Cells were collected and RNA was extracted using the RNeasy Mini Kit. Total RNA was reverse transcribed and quantitative real-time PCR was performed to assess mRNA levels of different HDACs. GAPDH was used as an internal control. The data are shown as relative mRNA level normalized to the level in scrambled control, which was set to 1. Data are shown as mean±s.d., n=3 replicates for each group. See Fig. 3a for measurement of surface CXCR4 expression in CB CD34^{+} cells. **p<0.01. ***p<0.001.

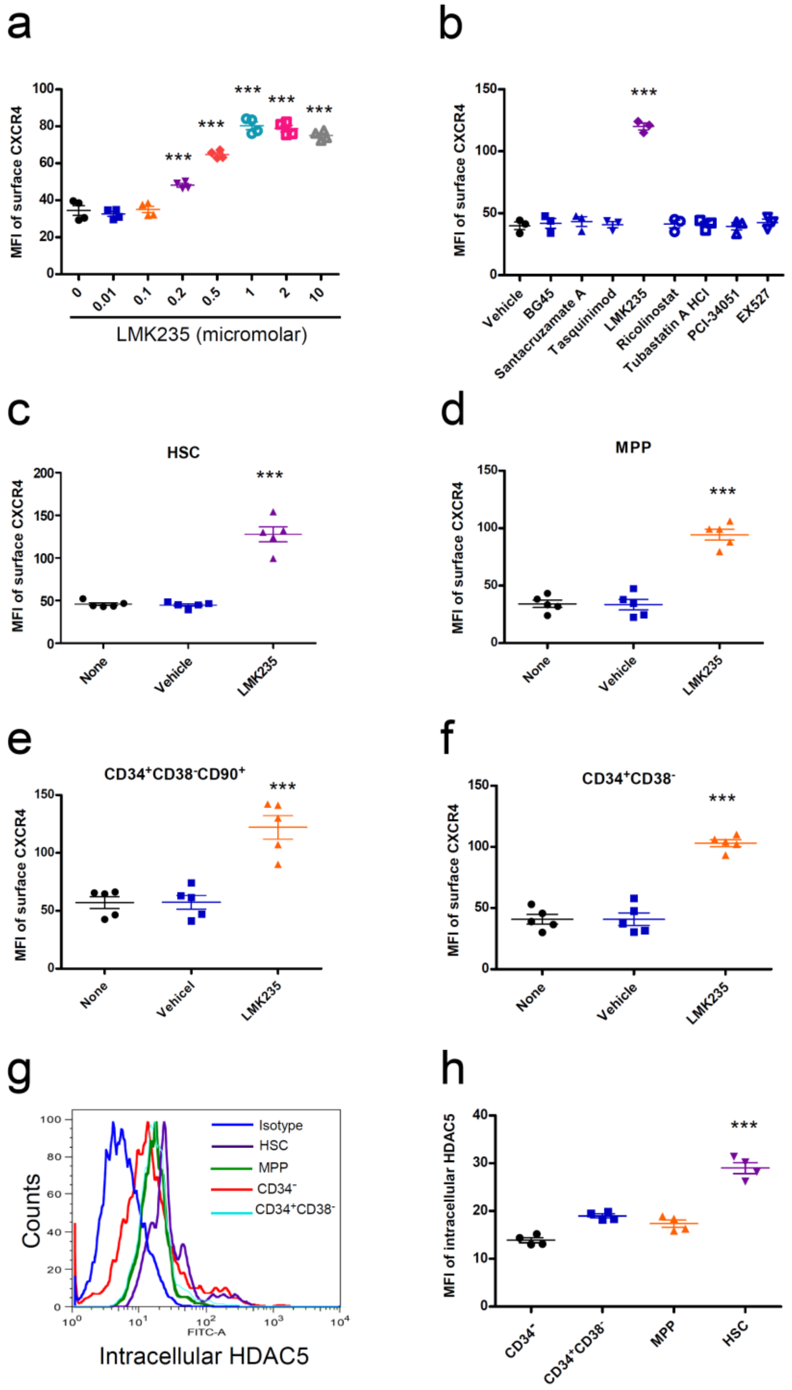

Supplementary Figure 3. Inhibition of HDAC5 promotes CXCR4 expression

(**a**) Quantification of MFI of surface CXCR4 of human CB CD34+ cells treated with vehicle or HDAC inhibitor M344 for different concentrations (μM). Data pooled from two independent experiments are shown (n=4, one-way ANOVA). (**b**) Quantification of MFI of surface CXCR4 of human CB CD34+ cells after treating cells for 16 hours with vehicle or different HDAC inhibitors. BG45 (HDAC1 and HDAC3 inhibitor), Santacruzamate A (HDAC2 inhibitor), Tasquinimod (HDAC4 inhibitor), LMK235 (HDAC5 inhibitor), Ricolinostat (HDAC6 inhibitor),

Tubastatin A HCl (HDAC6 inhibitor), PCI-34051 (HDAC8 inhibitor), EX527 (SIRT1 inhibitor) were used. Data pooled from three independent experiments are shown (n=3, one-way ANOVA). (**c-f**) Quantification of MFI of surface CXCR4 of human CB HSCs, MPPs, $CD34^+CD38^-CD90^+$ and $CD34^+CD38^-$ cells treated with vehicle or LMK235. None indicates the group without any treatment. Data pooled from five independent experiments are shown (n=5, one-way ANOVA). (**g**) Histogram of intracellular HDAC5 levels of human CB HSCs, MPPs, $CD34^+CD38^-$ and $CD34^-$ cells. Representative histogram from three independent experiments is shown. (**h**) Quantification of MFI of intracellular HDAC5 levels of human CB HSCs, MPPs, $CD34^+CD38^-$ and $CD34^-$ cells. Data pooled from two independent experiments are shown (n=4, one-way ANOVA). ***$p<0.001$.

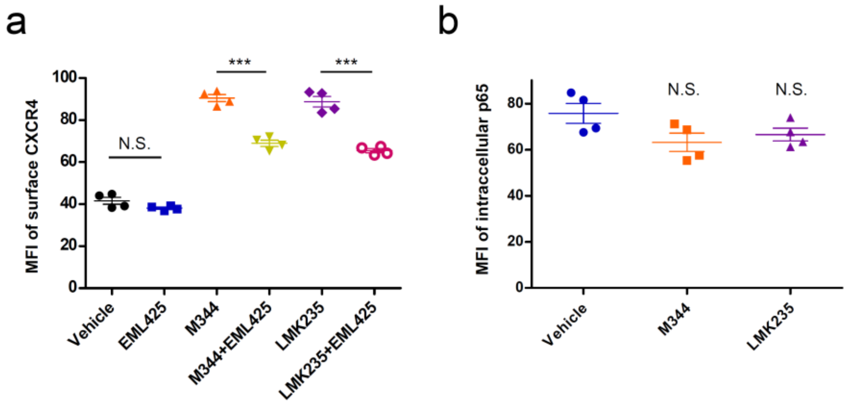

Supplementary Figure 4. Inhibition of HDAC5 enhances histone and p65 acetylation

(**a**) Mean fluorescence intensity (MFI) of surface CXCR4 in vehicle, EML425 (50 μM), M344, M344+EML425, LMK235, LMK235+EML425 treated human CB $CD34^+$ cells, as assessed by flow cytometry. Data pooled from two independent experiments are shown (n=4, one-way ANOVA). (**b**) Mean fluorescence intensity (MFI) of intracellular p65 levels in vehicle, M344 or LMK235 treated human CB $CD34^+$ cells, as assessed by flow cytometry. Data pooled from two independent experiments are shown (n=4, one-way ANOVA). Data are shown as dot plots (mean±s.e.m.). For all panels, ***p<0.001, N.S. indicates not significant.

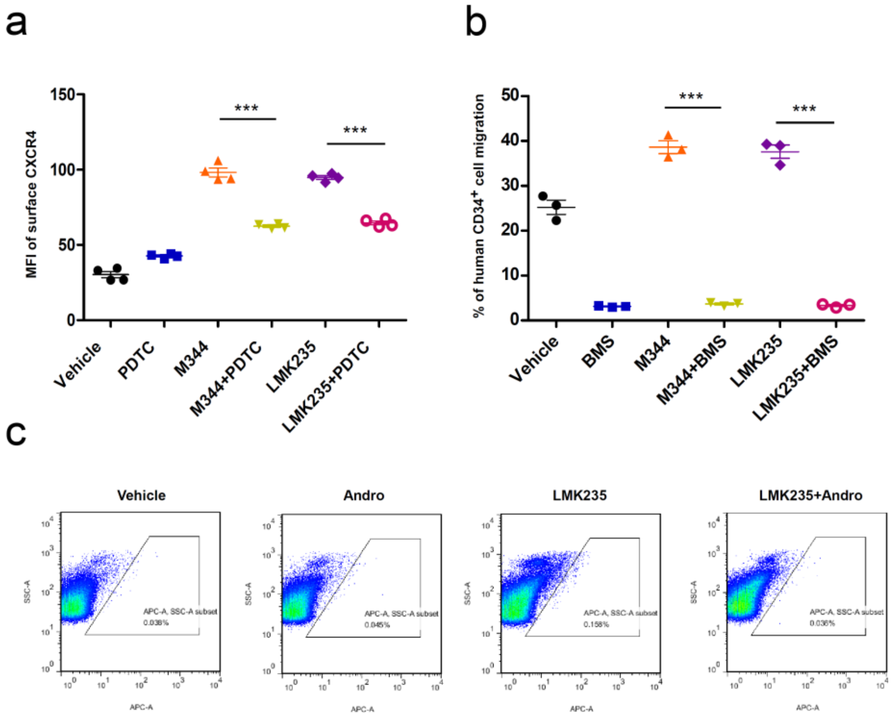

Supplementary Figure 5. NF-κB signaling pathway is involved in HDAC5 mediated CB HSC homing

(**a**) Mean fluorescence intensity (MFI) of surface CXCR4 in vehicle, Pyrrolidinedithiocarbamate ammonium (PDTC, 20 μM), M344, M344+PDTC, LMK235, LMK235+PDTC treated human CB $CD34^{+}$ cells, as assessed by flow cytometry. Data pooled from two independent experiments are shown (n=4, one-way ANOVA). Data are shown as dot plots (mean±s.e.m.), ***p<0.001. (**b**) The cells were cultured in the presence of vehicle, BMS345541 (BMS, 10 μM), M344, M344+BMS, LMK235, LMK235+BMS for 16 hours and then allowed to migrate towards 50 ng/mL SDF-1 for 4 hours. Data pooled from three independent experiments are shown (n=3, one-way ANOVA). Data are shown as dot plots (mean±s.e.m.), ***p<0.001. **(c)** Vehicle, Andrographolide (Andro, 10 μM), LMK235 or LMK235+Andro treated CB $CD34^{+}$ cells (500,000) were intravenously injected into each sublethally irradiated (350 cGy) NSG mouse. The percentage of human $CD45^{+}$ cells in bone marrow was determined after 24 hours. Representative pseudocolor plot is shown.

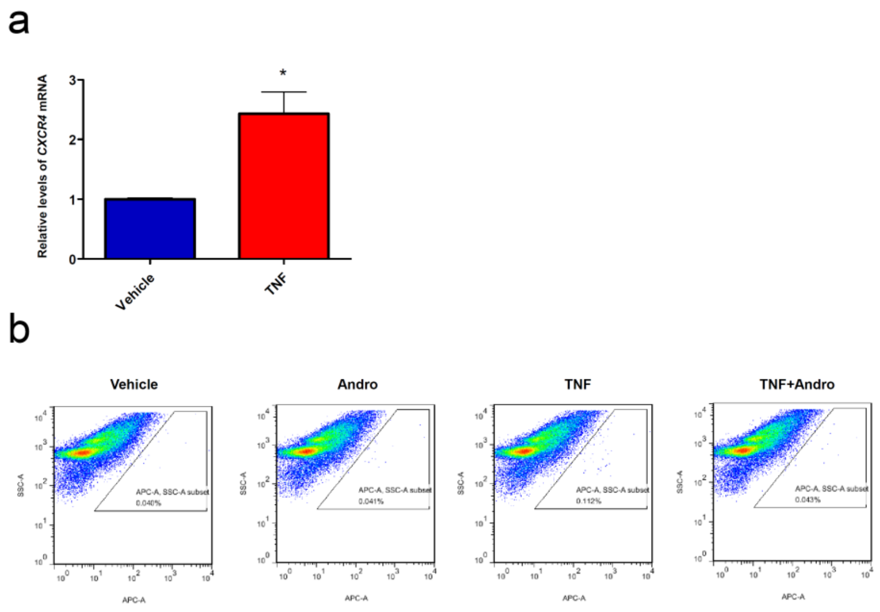

Supplementary Figure 6. TNFα treatment results in significantly enhanced HSC homing

(**a**) *CXCR4* mRNA levels in vehicle or TNFα treated human CB CD34$^+$ cells as assessed by quantitative RT-PCR. Data pooled from two independent experiments are shown (n=6, t test, *p<0.05). **(b)** Vehicle, Andrographolide (Andro, 10 μM), TNFα (TNF, 100 ng/mL), or TNF+Andro treated CB CD34$^+$ cells (500,000) were intravenously injected into each sublethally irradiated (350 cGy) NSG mouse. The percentage of human CD45$^+$ cells in bone marrow was determined after 24 hours. Representative pseudocolor plot is shown.

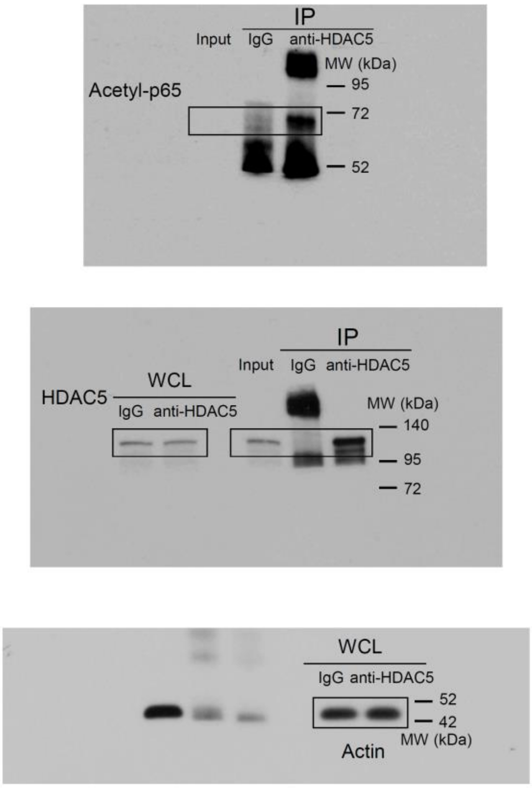

Supplementary Figure 7. Uncropped images of western blots in Fig. 6e.
